# Extended-spectrum beta-lactamase-producing *Pseudomonas aeruginosa* in camel in Egypt: potential human hazard

**DOI:** 10.1186/s12941-017-0197-x

**Published:** 2017-03-31

**Authors:** Mahmoud Elhariri, Dalia Hamza, Rehab Elhelw, Sohad M. Dorgham

**Affiliations:** 1 Department of Microbiology, Faculty of Veterinary Medicine, Cairo University, PO Box 12211, Giza, Egypt; 2 Department of Zoonoses, Faculty of Veterinary Medicine, Cairo University, PO Box 12211, Giza, Egypt; 3 Department of Microbiology and Immunology, National Research Centre, Giza, Egypt

**Keywords:** Camel, ESBL, *P. aeruginosa*, Resistance genes, Livestock, Egypt

## Abstract

**Background:**

The rapid increase of extended-spectrum beta-lactamase (ESBL) producing bacteria are a potential health hazard. Development of antimicrobial resistance in animal pathogens has serious implications for human health, especially when such strains could be transmitted to human. In this study, the antimicrobial resistance due to ESBL producing *Pseudomonas aeruginosa* in the camel meat was investigated.

**Methods:**

In this study meat samples from 200 healthy camels at two major abattoirs in Egypt (Cairo and Giza) were collected. Following culture on cetrimide agar, suspected *P. aeruginosa* colonies were confirmed with a Vitek 2 system (bioMe´rieux). *P. aeruginosa* isolates were phenotypically identified as ESBL by double disk synergy test. Additionally antimicrobial susceptibility testing of ESBL producing *P*. *aeruginosa* isolates were done against 11 antimicrobial drugs and carried out by disk diffusion method. The ESBL genotypes were determined by polymerase chain reaction according to the presence of the *bla*_PER-1_, *bla*_CTX-M_, *bla*_SHV_, and *bla*_TEM_.

**Results:**

*Pseudomonas aeruginosa* was isolated from 45 camel meat sample (22.5%). The total percentage of ESBL producing *P. aeruginosa* was 45% (21/45) from camel meat isolates. Antibiogram results revealed the highest resistance was for c, ceftriaxone and rifampicin followed by cefepime and aztreonam. The prevalence rates of β-lactamase genes were recorded (*bla*_PER-1_ 28.5%, *bla*_CTX-M_ 38%, *bla*_SHV_ 33.3% and *bla*_TEM_ 23.8%).

**Conclusions:**

This study illustrates the presence of high rates of ESBL-*P. aeruginosa* in camels that represents an increasing alarming for the risk of transmission to human and opens the door for current and future antibiotics therapy failure. Livestock associated ESBL-*P. aeruginosa* is a growing disaster, therefore, attention has to be fully given to livestock associated ESBL-bacteria which try to find its way to human beings.

## Background

The increasing resistance of potentially pathogenic bacteria to multiple conventional antibiotics is an urgent problem in global public health [1]. *Pseudomonas aeruginosa* is one of the major causes of diseases such as otitis, mastitis, endometritis, hemorrhagic pneumonia and urinary tract infections in both livestock and companion animals [2–4]. The multiple-drug-resistant (MDR) *Pseudomonas* can be transmitted from different sources to humans and also to the environment through horizontal gene, the emergence and occurrence of MDR *P. aeruginosa* strains are growing in the world, leading to limited therapeutic options [5, 6].

The prevalence of MDR enterobacteriaceae in slaughterhouses, including swine and poultry environments, has been reported in several studies building a growing alarm about their effect on animal and human health [7, 8]. Recently encountered the emergence of livestock associated ESBL-producing *P. aeruginosa* in cow, poultry and pigs [9, 10].

In Middle East region, camels are important in the livestock economy by their adaptability to adverse environmental conditions and naturally resistant to most of the diseases commonly affecting livestock [11]. Camels provide milk, meat, wool, hides, and skin, and their dung is used for fires [12]. They are acting one of major and good sources of protein and income for developing countries.

Transmission of ESBL-producing gram-negative bacteria between food-producing animals and humans via direct contact or meat is supposed [13]. Accordingly, livestock associated ESBL-producing gram-negative bacteria become new alarm for emerging infectious pathogens to human and animals.

This study aimed to investigate the role of camels as a risky reservoir and disseminating carrier of ESBL producing *P. aeruginosa* especially as this micro-organism has the ability of producing multidrug resistant enzymes that could be easily disseminated in the community via livestock products, particularly through direct contact and/or consumption of meat as well as other infected animal products.

## Methods

### Camel meat samples

This study was carried out on 200 apparently healthy camels. These camels were selected from two major abattoirs (Cairo and Giza) from April to September 2015. Meat samples from slaughtered camels in the abattoirs were collected in sterile containers and sent on ice to the microbiology department laboratory.

### Meat samples preparation

Twenty-five grams of the collected meat samples were weighed and transferred to sterile flasks containing 100 ml of phosphate buffer saline (PBS). Samples were homogenized using a meat grinder under aseptic conditions.

### Isolation and identification of *P. aeruginosa*

Two hundred meat samples were cultured into cetrimide agar, the plates were incubated aerobically at 37 °C for 24 h. The putative colonies were examined for their colonial morphology and microscopically according to Quinn et al. [14], the purified isolates of *P. aeruginosa* were finally confirmed with a Vitek 2 system (bioMe´rieux) which is rapid and reliable identification method.

### Phenotypic detection of ESBL by double-disk synergy test method (DDST)

ESBL production in *P. aeruginosa* was identified by the double disk synergy test (DDST) as described by Jarlier [15]. Mueller–Hinton agar was inoculated with standardized inoculum (corresponding to 0.5 McFarland tube) using a sterile cotton swab. An Augmentin (20 μg amoxicillin and 10 μg of clavulanic acid-AMC) disk was inserted in the center of the plate and test disks of 3rd generation cephalosporins (ceftazidime-CAZ 30 μg, ceftriaxone-CRO 30 μg, cefotaxime-CTX 30 μg) and aztreonam (ATM 30 μg) disks were placed at 20 mm distance (center to center) from the amoxicillin clavulanic acid disk. The plate was incubated overnight at 37 °C. Enhancement of the zone of inhibition of any one of the four drug disks toward amoxicillin–clavulanic acid suggested the presence of extended-spectrum beta-lactamases.

Escherichia coli 25922 was used as a negative control for the ESBL and *P. aeruginosa* ATCC 27853 was used as a control strain for a positive ESBL.

### Antimicrobial susceptibility testing

Antibiotic susceptibility tests were performed for ESBL producing *P. aeruginosa* isolates by using the standard disc diffusion method (Kirby–Bauer) on Mueller–Hinton agar plates. The standard procedures of the CLSI, 2015 and British Society for Antimicrobial Chemotherapy (BSAC) disc diffusion method were strictly followed [16, 17], accordingly the antimicrobial susceptibility of *P. aeruginosa* strains were tested against 11 antimicrobial drugs: aztreonam (30 μg), ceftazidime (30 μg), ceftriaxone (30 μg), cefepime (30 μg), amikacin (30 μg), gentamicin (10 μg), ciprofloxacin (5 μg), rifamycin (30 µg), imipenem (10 μg), meropenem (1 μg) and sulphamethoxazole/trimethoprim (25 µg). *E. coli* ATCC 25922 and *P. aeruginosa* ATCC 27853 were used as quality controls.

### DNA extraction

The whole genomic DNA from ESBL resistant *P. aeruginosa* strains totaling 21 was extracted using QIAamp Mini Kit (QIAGEN, Hombrechtikon, Switzerland) according to the manufacturer’s instructions.

### Molecular detection of ESBL-encoding genes

PCRs for detection of the *bla*_PER-1_, *bla*_CTX-M_, *bla*_SHV_ and *bla*_TEM_ genes. Specific primers for amplifying the selected genes by PCR are shown in Table 1.

**Table 1:** Primers used for detection of resistance genes

Target gene	Primer sequence (5′–3′)	Product size (bp)	References
*bla*_PER-1_	F: ATGAATGTCATTATAAAAGC	926	[18]
	R: TTAATTTGGGCTTAGGG		
*bla*_CTX-M_	F: GCGATGGGCAGTACCAGTAA	392	
	R: TTACCCAGCGTCAGATTCCG		
*bla*_SHV_	F: TCAGCGAAAAACACCTTG	472	[19]
	R: TCCCGCAGATAAATCACCA		
*bla*_TEM_	F: ATGAGTATTCAACATTTCCG	861	[20]
	R: TTACCAATGCTTAATCAGTGAG		

One uni plex PCR for detection of *bla*_TEM_ gene and two multiplex PCR were conducted for the (*bla*_PER-1_ and *bla*_CTX-M_) and another one for (*bla*_SHV_) gene

The reaction mixture consisted of 25 µl Platinum™ Hot Start PCR Master Mix (Invitrogen™), 1 µl DNA extract, 0.5 µl of each primer in the concentration of (20 pmol) and nuclease free water up to 50 µl. The cycling conditions included denaturation for 10 min at 95 °C, amplification for 30 cycles of 30 s at 95 °C, 1 min at 55 °C, and 1 min at 72 °C, and extension for 10 min at 72 °C. The PCR products were resolved by electrophoresis on 1.5% (wt/vol) agarose gels (QIAGEN, Hombrechtikon, Switzerland).

## Results

### Isolation and identification of *P. aeruginosa*

Forty-five isolates (22.5%) from meat samples of 200 camels were produced characteristic growth features of *Pseudomonas* species on the cetrimide agar medium. The isolates were obtained as one specific colony per camel meat sample. The isolates were confirmed as by Vitek 2 system (bioMe´rieux).

### Phenotypic detection of ESBL by double-disk synergy test method (DDST)

Phenotypic detection of ESBL by DDST revealed that 21 *P. aeruginosa* isolates. Accordingly, a total percentage of ESBL was detected in overall, 45% (21/45) from camel meat isolates.

### Antimicrobial susceptibility testing

The results of the antimicrobial susceptibility testing for the 21 ESBL *P. aeruginosa* shows a high-level resistance (100%) to ceftazidime, ceftriaxone and rifampicin followed by cefepime (95.2%) and aztreonam (76.1%). The susceptibilities of the isolates to meropenem, amikacin, imipenem, gentamicin and ciprofloxacin were 85.8, 81.0, 76.2, 71. 4 and 66.7 respectively (Table 2).

**Table 2:** Antibiotic resistance pattern of 21 *Pseudomonas aeruginosa* isolates

Antibiotic	No (%) resistant	No (%) sensitive
Amikacin (30 μg)	4 (19.0)	17 (81.0)
Imipenem (10 μg)	5 (23.8)	16 (76.2)
Gentamicin (10 μg)	6 (28.5)	15 (71.4)
Ciprofloxacin (5 μg)	7 (33.3)	14 (66.7)
Sulphamethoxazole/trimethoprim (25 µg)	8 (38.0)	13 (61.9)
Rifampicin (30 μg)	21 (100)	–
Ceftriaxone (30 μg)	21 (100)	–
Cefepime (30 μg)	20 (95.2)	1 (4.7)
Aztreonam (30 μg)	15 (76.1)	6 (28.5)
Meropenem (1.0 μg)	3 (14.2)	18 (85.8)
Ceftazidime (30 μg)	21 (100)	–

### Molecular detection of ESBL-encoding genes

PCR screening of genes encoding ESBL revealed the amplification of *bla*_PER-1_, *bla*_CTX-M_, *bla*_SHV_ and *bla*_TEM_ genes in all tested isolates except three isolates coded p-4, -11, and -18 did not harbor any of ESBL genes (Table 3).

**Table 3:** ESBL resistance genes percent and antibiotic pattern of multiple drug resistant strains

Isolates	Resistance gene pattern				Antimicrobial resistance	Antibiotic pattern
	*bla*_PER-1_	*bla*_CTX-M_	*bla*_SHV_	*bla*_TEM_		
*P*-1		+		+	CAZ, CRO, RD, FEP, ATM, AK, CN, CIP	MDR
*P*-2	+	+	+		CAZ, CRO, RD, FEP, ATM, MEM	MDR
*P*-3		+		+	CAZ, CRO, RD, FEP, ATM, CIP	MDR
*P*-4					CAZ, CRO, RD, FEP, ATM, CN	MDR
*P*-5		+			CAZ, CRO, RD, FEP, IPM	MDR
*P*-6	+				CAZ, CRO, RD, FEP, ATM, SXT	MDR
*P*-7		+	+		CAZ, CRO, RD, FEP, ATM, IPM	MDR
*P*-8			+	+	CAZ, CRO, RD, FEP, ATM,AK, CIP	MDR
*P*-9	+		+		CAZ, CRO, RD, FEP, ATM, MEM, CIP, SXT	MDR
*P*-10	+				CAZ, CRO, RD, FEP, CN, CIP	MDR
*P*-11					CAZ, CRO, RD, FEP, ATM, CIP	MDR
*P*-12				+	CAZ, CRO, RD, FEP, AK, CIP	MDR
*P*-13	+	+			CAZ, CRO, RD, FEP, CN	MDR
*P*-14			+		CAZ, CRO, RD, FEP, IPM, SXT	MDR
*P*-15				+	CAZ, CRO, RD, FEP, AK, CN	MDR
*P*-16		+			CAZ, CRO, RD, FEP, ATM, SXT	MDR
*P*-17		+	+		CAZ, CRO, RD, FEP, ATM, SXT	MDR
*P*-18					CAZ, CRO, RD, FEP, IPM, CN	MDR
*P*-19				+	CAZ, CRO, RD, FEP, SXT	MDR
*P*-20	+				CAZ, CRO, RD, FEP, ATM, IPM, SXT	MDR
*P*-21			+		CAZ, CRO, RD, MEM, SXT	MDR
%	(6/21) 28.5	(8/21) 38	(7/21) 33.3	(6/21) 28.5		

*MDR* Multiple drug resistant

Furthermore, eight out of 21 ESBL-positive isolates had the *bla*_CTX-M_ (38%), seven had *bla*_SHV_ gene (33.3), and six carried *bla*_TEM_ and *bla*_PER_ genes (28.5).

## Discussion

Extended-spectrum beta lactamase-producing bacteria are one of the fastest emerging resistance problems worldwide. ESBL-producing bacteria were observed in human medical practice, the observation of these bacteria in companion animals and the increase in livestock has initiated monitoring studies concentrating on livestock [13, 21].

Livestock may be an important vehicle for the community-wide dissemination of antimicrobial-resistant *Enterobacteriaceae,* also *P. aeruginosa* especially ESBL-producing type isolates have been found in increasing numbers in food-producing animals [9, 10, 22].

Accordingly to the hypothesis that animals might become infection sources or even natural persistent sources acting as risky reservoirs of infection leading to the spread of these bacteria specifically multidrug resistant types in community [23]. There are essential needs for monitoring or surveillance studies incorporating veterinary medicine to identify transmissible pathogens to human and its risk factors.

The dromedary camel is a good source of meat, especially in areas where the climate adversely affects the performance of other meat animals. This is because of the unique physiological characteristics of camels [24]. The interest for camel meat seems by all accounts to be expanded because of wellbeing reasons, as it contains less fat and in addition less cholesterol and generally high polyunsaturated unsaturated fats than other animal’s meat [25].

To the best of our knowledge, this is the first time that an attempt has been made to determine ESBL *P. aeruginosa* resistance in camel meat. In this study, the prevalence of *P. aeruginosa* in camel was determined about 22.5% (45/200). This percent is so higher to the encountered investigations on fish and cow in Switzerland and Nigeria [9, 26]. Even though *P. aeruginosa* is ubiquitous, the prevalence of its isolation among camel isolates in this study was lower compared to the presence rate of *E. coli* that isolated from camel in Saudi Arabia with 26.0% [27].

Class A (ESBLs) are typically identified in *P. aeruginosa* isolates and showing resistance to the extended-spectrum cephalosporin (ESCs) [28], this resistance is often due to the production of β-lactamases. Clinically, ESBLs are generally encoded by plasmid-mediated *bla* genes; three major clinically relevant β-lactamase genes are *bla*_SHV_, *bla*_TEM_ and *bla*_CTX-M 2, 4_ [29].

The total percentage of ESBL producing *P. aeruginosa* was 45% (21/45) from camel meat isolates by DDST, accordingly, the most frequently β-lactamase-genes detected in this isolates were *bla*_CTX-M_ (38%), followed by *bla*_SHV_ (33.3%) and *bla*_TEM_, *bal*_PER-1_ (28.5%).

CTX-M enzymes have now become the most widespread type of ESBL worldwide and represent the wide dissemination of particular plasmids or bacterial clones [30]. On the other hand, *bla*_PER-1_ were found to be the most prevalent type of β-lactamase-encoding genes in *Acinetobacter baumannii* in Egypt [31] and this suggests the horizontal transfer of this gene.

In the present study antibiogram of ESBL producing *P. aeruginosa* showed high-level resistance (100%) to ceftazidime, ceftriaxone and rifampicin followed by cefepime (95.2%) and aztreonam (76.1%).The susceptibilities of the isolates to meropenem, amikacin, imipenem, gentamicin, ciprofloxacin and amikacin were 85.8, 81.0, 76.2, 71.5 and 67% respectively (Table 2).

*Pseudomonas aeruginosa* and other gram negative bacteria with ESBLs contain other β-lactamases that makes difficult the phenotypic detection of ESBL [32], this issue need further investigation.

Many investigators focus on MDR *P. aeruginosa* from human. *P. aeruginosa* are multi-drug resistant to amikacin (17.25%), ciprofloxacin (27.59%), ceftriaxone varied from 51.00 to 73.00% and all the strains were susceptible to imipenem (20.69%) [33]. High resistance to aminoglycosides had been reported in Malaysia [34]. Similarly higher rates of resistance to fluoroquinolones such as ciprofloxacin (40.5%) had been reported in a study done in India. Lesser rate of resistance to ceftriaxone (40%) had been reported in Andhra Pradesh, India [35].

The presence of high resistance profile by camel *P. aeruginosa* isolates my attributed antibiotics used in management of this animals or natural resistance of camel that suites it as a risk reservoir for such pathogens.

## Conclusion

The present study demonstrates, for the first time in Egypt, the presence a high rate of ESBL-*P. aeruginosa* in camels, The abundance MDR *P. aeruginosa* from camel meat in this study suggests a potential risk of human susceptibility to such pathogen group which remodels the epidemiology of antibiotic resistance. Urgent control measures are necessary to be applied to restrict the continuous abuse of antibiotics, especially in livestock production.

## Abbreviations

ESBL: extended-spectrum beta-lactamase
MBL: metallo-β-lactamases
DDST: double disk synergy test
AST: antimicrobial susceptibility testing
PCR: polymerase chain reaction
MDR: multiple-drug-resistant
HGT: horizontal gene transfer
DDST: double-disk synergy test method
CLSI: the National Committee for Clinical Laboratory Standards
CAZ: ceftazidime
AK: amikacin
CRO: ceftriaxone
FEP: cefepime
ATM: aztreonam
MEM: meropenem
CIP: ciprofloxacin
SXT: sulphamethoxazole/trimethoprim
IPM: imipenem
CN: gentamicin
RD: rifampicin
